# The ferroptosis inducer RSL3 triggers interictal epileptiform activity in mice cortical neurons

**DOI:** 10.3389/fncel.2023.1213732

**Published:** 2023-06-15

**Authors:** Michela Giustizieri, Sara Petrillo, Jessica D’Amico, Caterina Torda, Andrea Quatrana, Federico Vigevano, Nicola Specchio, Fiorella Piemonte, Enrico Cherubini

**Affiliations:** ^1^European Brain Research Institute (EBRI)-Rita Levi-Montalcini Foundation, Rome, Italy; ^2^Muscular and Neurodegenerative Diseases Laboratory, Research Area of Neurological Sciences and Rehabilitation Medicine, Bambino Gesù Children’s Hospital, IRCCS, Rome, Italy; ^3^Neurology Unit, Research Area of Neurological Sciences and Rehabilitation Medicine, Bambino Gesù Children’s Hospital, IRCCS, Rome, Italy; ^4^Clinical and Experimental Neurology, Bambino Gesù Children’s Hospital, IRCCS, Full Member of European Reference Network on Rare and Complex Epilepsies (EpiCARE), Rome, Italy

**Keywords:** neurodevelopmental disorders, epilepsy, interictal discharges, RSL3, Ferroptosis, E/I unbalance, GABAergic and Glutamatergic signaling, disinhibition

## Abstract

Epilepsy is a neurological disorder characterized by recurrent seizures, which result from excessive, synchronous discharges of neurons in different brain areas. In about 30% of cases, epileptic discharges, which vary in their etiology and symptomatology, are difficult to treat with conventional drugs. Ferroptosis is a newly defined iron-dependent programmed cell death, characterized by excessive accumulation of lipid peroxides and reactive oxygen species. Evidence has been provided that ferroptosis is involved in epilepsy, and in particular in those forms resistant to drugs. Here, whole cell patch clamp recordings, in current and voltage clamp configurations, were performed from layer IV principal neurons in cortical slices obtained from adult mouse brain. Application of the ferroptosis inducer RAS-selective lethal 3 (RSL3) induced interictal epileptiform discharges which started at RSL3 concentrations of 2 μM and reached a plateau at 10 μM. This effect was not due to changes in active or passive membrane properties of the cells, but relied on alterations in synaptic transmission. In particular, interictal discharges were dependent on the excessive excitatory drive to layer IV principal cells, as suggested by the increase in frequency and amplitude of spontaneously occurring excitatory glutamatergic currents, possibly dependent on the reduction of inhibitory GABAergic ones. This led to an excitatory/inhibitory unbalance in cortical circuits. Interictal bursts could be prevented or reduced in frequency by the lipophilic antioxidant Vitamin E (30 μM). This study allows identifying new targets of ferroptosis-mediated epileptic discharges opening new avenues for the treatment of drug-resistant forms of epilepsy.

## Introduction

The term “Ferroptosis” introduced by Dixon et al. (2012) in 2012, refers to a newly discovered non-apoptotic, regulated, iron-dependent cell death, characterized by accumulation of intracellular iron ions. Iron is involved in many redox reactions: in its ferrous form (Fe^2+^), through the Fenton reaction, it catalyzes the conversion of H_2_O_2_ into highly reactive hydroxyl radicals and iron’s ferric form (Fe^3+^) (Winterbourn, 1995). Hydroxyl radicals reacting with oxygen lead to the production of Reactive Oxygen Species (ROS). Among ROS, hydroxyl radicals are the strongest oxidant, able to interfere with a wide range of biological substrates such as proteins, lipids and nucleic acids (Yang and Stockwell, 2008; Friedmann Angeli et al., 2014; Stockwell et al., 2017).

Ferroptosis-induced cell death is tightly controlled by numerous metabolic pathways (Stockwell et al., 2020; Stockwell, 2022), and is driven by peroxidation of phospholipids (Jang et al., 2021), the major components of cell membranes. In physiological conditions, cell death is prevented by glutathione-peroxidase-4 (GPX4), an enzyme known to catalyze the detoxification of phospholipid hydroperoxides (PLOOHs) by their reduction into phospholipid alcohols (Ursini et al., 1982, 1985; Seiler et al., 2008; Liang et al., 2022).

Ferroptosis has been demonstrated to occur in a variety of pathological conditions including organ’s injury, infectious, autoimmune diseases, cancer and neurological disorders (for reviews see: Kahn-Kirby et al., 2019; Li et al., 2020; Tang et al., 2020; Liang et al., 2022; Stockwell, 2022). Among the latter of particular interest are neurodevelopmental disorders such as epilepsies and associated cognitive impairment caused by excessive oxidative stress (Lin et al., 2020). Giving the critical role of iron in driving lipid peroxidation, it is not surprising that iron overload following bleeding with consequent hemolysis and accumulation of hemoglobin in the neocortex is a common cause of hemorrhagic post-stroke and post-traumatic epilepsy (Mori et al., 1990; Ikeda, 2001).

Using an *in vivo* 3D two-photon imaging approach to study Fe^2+^ dynamics and unveil the presence of ferroptosis, Shao et al. (2021) have been able to detect abnormal elevations of ferrous ions (Fe^2+^) in the epileptic mouse brain, an effect that could be reduced by dihydroartemisin (DHA), a compound that plays a critical role in regulating iron homeostasis and in reducing seizure activity.

Hallmarks of ferroptosis have been detected in several animal models of epilepsy (Kahn-Kirby et al., 2019; Mao et al., 2019; Du et al., 2022; Wang et al., 2022) and, noteworthy, several ferroptosis inhibitors are able to prevent neuronal loss, to reduce seizure activity and to ameliorate cognitive impairment (Ye et al., 2019; Wang et al., 2022; Xie et al., 2022; Zhang et al., 2022).

The major challenge in epilepsy research is to find new approaches to prevent or reduce the occurrence of seizures and associated cognitive deficits, in particular those resistant to drugs, as an alternative strategy to epileptic focus neurosurgical resection, which is invasive and not always effective. Aim of the present study was to identify new therapeutic targets of ferroptosis-dependent epileptic discharges. In particular, we asked whether, by enhancing PLOOHs accumulation *via* direct inhibition of GPX4, the ferroptosis inducer RSL3 is able to enhance cell excitability and to trigger seizures activity in mouse cortical slices. RSL3 is a small molecule that directly inhibits GPX4 by binding the seleno-cysteine residue on the active site of the enzyme (Yang and Stockwell, 2008).

Here, we found that RSL3 generates, in a concentration-dependent way, interictal discharges which rely on an excitatory/inhibitory unbalance, consequent to the excessive excitatory drive to layer IV principal cells, possibly caused by disinhibition. We also found that the lipophilic antioxidant Vitamin E, is able to protect cell membranes by counteracting RSL3-mediated effects.

## Materials and methods

### Animals and ethical approval

All experiments were performed on C57BL/6J mice, in accordance with the Italian Animal Welfare legislation (D.L. 26/2014), implemented by the European Committee Council Directive. Experiments were approved by local veterinary authorities, the EBRI ethical committee and the Italian Ministry of Health (protocol F8BBD.N.HCD). All efforts were made to minimize animal suffering and to reduce the number of animal used.

### Cortical slices

Cortical slices were obtained from postnatal (P) day P30–P40 old animals (male and female), using a standard protocol (Schubert et al., 2001). Briefly, after being anesthetized with an intraperitoneal injection of a mixture of tiletamine/zolazepam (zoletyl, 80 mg/kg body weight) and xylazine (rompun, 10 mg/kg body weight), mice were decapitated. The brain was quickly removed from the skull and placed in ice-cold artificial cerebral-spinal fluid (ACSF) containing (in mM): sucrose 65, NaCl 85, glucose 10, KCl 2.5, NaH_2_PO_4_ 1.25, NaHCO_3_ 25, CaCl_2_ 0.5, MgCl_2_ 7, saturated with 95% O_2_ and 5% CO_2_. Coronal slices (300 μm thick) were cut with a vibratome and stored at room temperature (22–24°C) in a holding bath containing the same solution as above. After incubation for at least 1 h, an individual slice was transferred to a submerged recording chamber and continuously perfused at 33–34°C with oxygenated ACSF of the following composition (in mM): NaCl 130, glucose 10, KCl 3.5, NaH_2_PO_4_ 1.2, NaHCO_3_ 24, CaCl_2_ 3, MgCl_2_ 0.5, at a rate of 3 ml/min. For each experimental approach, data were collected from several slices (3–10) obtained at least from 3 different mice.

### Electrophysiology

Pyramidal cells in layer IV of the somatosensory barrel cortex (spiny neurons) were visualized with a 60× water immersed objective mounted on an upright microscope (Olympus CX23) equipped with differential interference contrast optics and an infrared video camera (Scientifica, UK). Whole-cell patch clamp recordings in voltage and current clamp mode were performed with a MultiClamp 700B amplifier (Axon Instruments, Sunnyvale, CA, USA). Patch electrodes were pulled from borosilicate glass capillaries (WPI, Florida, USA). They had a resistance of 3–5 MΩ when filled with an intracellular solution. Membrane potential values were not corrected for liquid junction potentials. Miniature GABA_A_-mediated inhibitory postsynaptic currents (mIPSCs) and AMPA-mediated excitatory postsynaptic currents (mEPSCs) were recorded in the presence of tetrodotoxin (TTX 1 μM), to block sodium currents and propagated action potentials, 6-cyano-7-nitroquinoxaline-2,3-dione (CNQX, 10 μM) or picrotoxin (100 μM), to block AMPA or GABA_A_ receptors, respectively. Two different intracellular solutions were used to record inhibitory and excitatory postsynaptic events as inward currents from the same holding potential (−70 mV). Miniature and spontaneous inhibitory postsynaptic currents (mIPSCs and sIPSCs) were recorded with an intracellular solution containing (in mM): K gluconate 70, KCl 70, HEPES 10, EGTA 0.2, MgCl_2_ 2, MgATP 4, MgGTP 0.3, Na-phosphocreatine 5; the pH was adjusted to 7.2 with KOH; the osmolarity was 295–300 mOsm (E_GABA_ −70 mV). Miniature and spontaneous AMPA-mediated excitatory postsynaptic currents (mEPSCs and sEPSCs) were recorded with an intracellular solution containing (in mM): K-gluconate 110, HEPES 40, NaCl 4, Mg-ATP 2, Na-GTP 0.3. The pH was adjusted to 7.2 with KOH; the osmolarity was 290–300 mOsm. For the Excitatory/Inhibitory balance experiments, an intracellular solution containing (in mM): cesium methanesulfonate 130, NaCl 5, HEPES 10, EGTA tetrasodium 1, MgCl_2_ 2, Mg-ATP 2, Na-GTP 0.5, phosphocreatine 10, was used. The holding membrane potential value was not corrected for the liquid junction potential of 10 mV (calculated with the Clampex software; Molecular Devices, Sunnyvale, CA, USA). The stability of the patch was checked by repetitively monitoring the input and series resistance during the experiments. Cells exhibiting >15% changes were excluded from the analysis. The series resistance was <20 MΩ and it was not compensated.

Pharmacologically isolated spontaneous AMPA-mediated excitatory postsynaptic currents (sEPSCs) or GABA_A_-mediated inhibitory postsynaptic currents (sIPSCs) were recorded in spiny neurons from a holding potential of -70 mV, in the presence of picrotoxin (100 μM) or 6-cyano-7- nitroquinoxaline-2,3-dione (CNQX, 10 μM), respectively.

Excitatory postsynaptic currents and IPSCs, evoked by stimulation of afferent fibers in layer V with a bipolar silver electrode (stimulation frequency: at 0.1 Hz; stimulus duration: 100 μs; stimulus intensity equal to that necessary to obtain synaptic responses equal to 50% of the maximal ones), were recorded as inward or outward currents at the reversal potentials of GABA (E_GABA_: −70 mV) or glutamate (E_Glu_: 0 mV), respectively. The respective areas underlying EPSCs and IPSCs were used to measure the Excitatory/Inhibitory balance (see Cellot and Cherubini, 2014). Paired responses, evoked by two stimuli 50 ms apart, were used to measure the paired-pulse ratio (PPR). Membrane potential values were corrected for a liquid junction potential of ∼4 mV.

Network activity involving the synchronous activation of neuronal ensembles, occurring simultaneously with interictal discharges recorded from layer IV spiny neurons in current clamp mode, was detected as field potentials by an extracellular electrode positioned in layer V, close to the apical dendrites of layer IV pyramidal neurons.

### Data analysis

Data were transferred to a computer hard disk after digitization with an A/D converter (Digidata 1550B, Molecular Devices). Data acquisition (digitized at 10 kHz and filtered at 1 kHz) was performed with pClamp 11.1 software (Molecular Devices, Sunnyvale, CA, USA). Input resistance and cells capacitance were measured online with the membrane test feature of the pClamp software. Possible RSL3-induced changes in passive and active membrane properties were assessed by measuring, in the presence of glutamatergic and GABAergic antagonists (DNQX, D-APV and picrotoxin), electrotonic potentials and cell firing induced in spiny neurons by hyperpolarizing and depolarizing current steps (10 pA, 50 ms duration), respectively. Spike’s threshold, amplitude and half width have been measured with clampfit 11.1 software.

Spontaneous and miniature EPSCs and IPSCs were analyzed with pClamp 11.1 (Molecular Devices, Sunnyvale, CA, USA). This program uses a detection algorithm based on a sliding template. The template did not induce any bias in the sampling of events because it was moved along the data trace by one point at a time and was optimally scaled to fit the data at each position. Cumulative distributions for inter event intervals and amplitudes were then constructed.

The E/I balance was assesses by measuring, in different experimental conditions, the ratio between the averaged area (pA*ms) underlying individual EPSCs and IPSCs recorded at the reversal potentials of GABA (E_GABA_) and glutamate (E_Glu_), respectively. The PPR was measured as the mean peak amplitude of the synaptic response evoked by the second stimulus over that evoked by the first one (the two stimuli were 50 ms apart).

### Statistical analysis

Statistical significance was tested using the paired Student *t*-test (OriginLab software). For data that did not follow a normal distribution, as in cumulative probability plots of frequency and amplitude excitatory and inhibitory synaptic activity, Kolmogorov–Smirnov’s test was used. A two way ANOVA test has been used to determine the effects of RSL3 and RSL3 plus Vitamin E on bursting activity of layer IV spiny neurons. A *p*-value < 0.05 was considered as statistically significant. Values are given as mean ± SEM.

### Drugs

Drugs were applied by gravity by changing the superfusion solution to one differing only in its content. The ratio of flow rate to bath volume ensured complete exchange within 1–2 min. Drugs used were: 5-Phosphononorvaline, AP-5, Norvaline, DL-2-Amino-5-phosphonovaleric acid (DL-AP5), and 6,7-dinitroquinoxaline-2,3-dione (DNQX) purchased from Ascent Scientific (Bristol, UK). Stock solutions were made in distilled water and then aliquoted and frozen at –20°C. DNQX was dissolved in Dimethyl sulfoxide (DMSO). The final concentration of DMSO in the bathing solution was 0.1%. At this concentration, DMSO alone did not modify the membrane potential, input resistance or the firing properties of neurons. RAS-selective lethal-3 (RSL-3) and Tetrodotoxin citrate were purchased from Tocris.

### Western blot

Cortical slices were homogenized (1:10 w/v) in RIPA buffer (50 mM Tris–HCl, pH 8.0, 150 mM NaCl, 12 mM deoxycholic acid, 0.5% Non-idet P-40, and protease and phosphatase inhibitors). 40 μg proteins were subjected to SDS PAGE on 4–12% denaturing gel and probed with GPX4 (1:1,000, Thermo Fisher Scientific, USA), and GAPDH (1:10,000, Sigma) as loading control. Immuno-reactive bands were detected using the Lite Ablot Extend Long Lasting Chemiluminescent substrate (Euroclone, Milan, Italy). Signals derived from appropriate HRP-conjugated secondary antibodies (Bethyl Laboratories, Montgomery, TX, USA) were captured by Chemi Doc™ XRS 2015 (Bio-Rad Laboratories, Hercules, CA, USA) and densitometric analysis was performed using Image Lab software (Version 5.2.1, Bio-Rad Laboratories).

## Results

In a first set of experiments performed in current clamp mode, we tested the effects of increasing concentrations of RSL3 on the firing properties of layer IV principal cells (held at −70 mV) in the barrel cortex of postnatal (P) P30-P40 male and female mice. Since we did not find any gender-related difference, data obtained from males and females have been pooled together.

**FIGURE 1:**
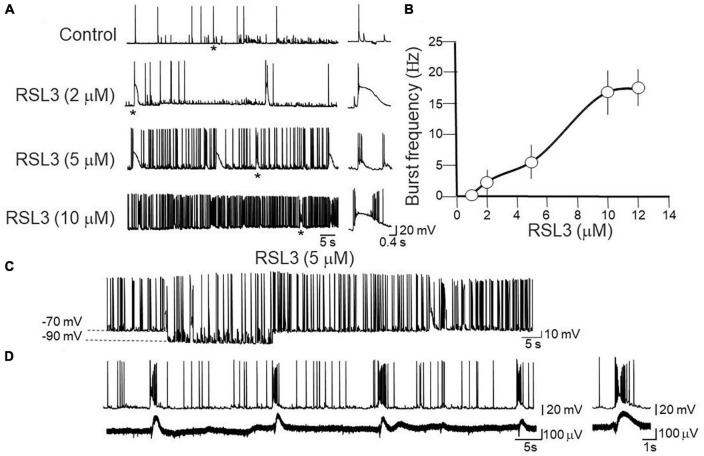
Dose-dependent induction of network-driven interictal bursts by RSL3. **(A)** Representative traces of spontaneously occurring action potentials and bursting activity recorded at –70 mV from layer IV cortical spiny neurons in control and 15-min after application of increasing concentrations of RSL3. Firing or bursting activity marked by an asterisk is shown on the right on an expanded time scale. **(B)** Dose-response curve of burst’s frequency induced by increasing concentrations of RSL3. RSL3 effects start at concentrations of 2 μM and reach a steady state at 10 μM. Each point is the mean of 8 samples from 3 animals. Bars represent the SEM. **(C)** Sample trace of spontaneously occurring bursting activity induced by RSL3 (5 μM) at two different holding potentials. Note that the bursting activity persisted by hyperpolarizing the membrane from –70 to –90 mV. **(D)** Sample trace of interictal bursts induced by RSL3 (5 μM), recorded simultaneously from a spiny neuron with a patch electrode (upper trace) and from a population ensemble (field potentials, lower trace) with an extracellular electrode positioned close to the apical dendrites of layer IV principal cell.

Application of RSL3 in the bath, induced within 15 min, an enhancement of spontaneous synaptic activity and the appearance of spontaneous bursts that persisted after washing out RSL3 for at least 30 min. A burst was defined as a transient sequence of two or more action potentials occurring at high frequency (>20 Hz; Zucca et al., 2017), often driven by a slow depolarizing after potential which allowed recruiting additional action potentials. Bursting activity varied in a dose-dependent way: it became detectable with RSL3 (2 μM), increased in frequency with higher concentrations of the drug and reached a steady state with RSL3 (10 μM) (Figures 1A, B).

In the CA3 region of the hippocampus two types of bursts have been identified: endogenous and network-driven ones (Hablitz and Johnston, 1981). According to the criteria established by Johnston and Brown (1981), in contrast to network-driven bursts whose frequency is independent on the membrane potential, endogenous ones are blocked when the membrane potential is moved toward more hyperpolarized values. In addition, in contrast to network-driven bursts, endogenous ones are not associated with concomitant extracellularly recorded field potentials, which reflect the synchronous activation of a neuronal ensembles. In our case, bursts induced by RSL3 were associated with population responses and they persisted when the membrane was hyperpolarized from −70 to −90 mV (Figures 1C, D). Therefore, they met the criteria for network driven events and the underlying paroxysmal depolarizing shifts for giant excitatory postsynaptic potentials (EPSPs), closely resembling those induced by kainate and other convulsants (Ben-Ari and Gho, 1988).

To further assess whether RSL3-induced increase in cell excitability was dependent on modifications of passive or active membrane properties of layer IV principal neurons, we measured first the resting membrane potential, cell capacitance and input resistance of these cells. As shown in Table 1, comparable values of mean resting membrane potential (V_rest_), cell capacitance (C_m_) and input resistance (R_in_) were detected in layer IV spiny neurons from controls and RSL3-treated (5 μM) slices.

**TABLE 1:** Resting membrane potentials, cell capacitance and input resistance measured in layer IV spiny neurons from control and RSL3-treated slices.

	N	Resting membrane potential (Vrest)	Cell capacitance (Cm)	Input resistance (Rin)
Control	12	−69.0 ± 0.8 mV	91.5 ± 2.0 pF	188.0 ± 5.0 MΩ
RSL3 (5 μM)	12	−68.9 ± 0.4 mV	93.0 ± 4.0 pF	186.0 ± 2.4 MΩ

**FIGURE 2:**
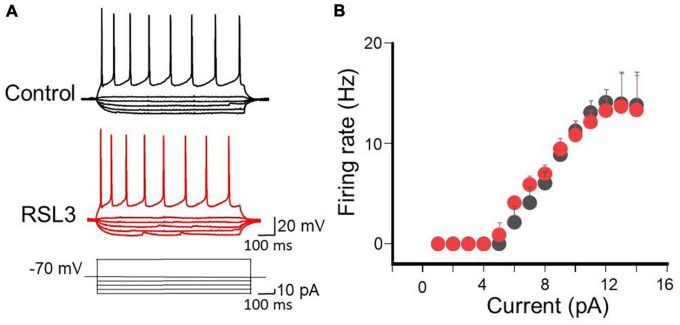
RSL3 does not alter membrane conductances of spiny neurons. **(A)** Sample traces of eletrotonic potentials and firing activity induced in layer IV spiny neurons (in the presence of AMPA, NMDA and GABA receptor antagonists), in the absence (black) or in the presence of RSL3 (5 μM, red), by hyperpolarizing an depolarizing current pulses, respectively. **(B)** Each point represents the mean firing rate of spiny neurons evoked by a depolarizing current step of 40 pA from a holding potential of –70 mV, in the absence (black, *n* = 6/3animals) or in the presence (red, *n* = 6/3animals) of RSL3 (5 μM). Bars represent SEM. Note complete overlapping of black and red points.

Possible RSL3-induced changes in active membrane properties were analyzed by measuring, in the presence of CNQX (10 μM), DL-APV (50 μM) and picrotoxin (100 μM) to block synaptic transmission, electrotonic potentials and the firing rate evoked by respective hyperpolarizing or depolarizing current pulses of increasing amplitude (10 pA). As shown in Figure 2, no differences in electrotonic potentials evoked by hyperpolarizing current pulses and action potentials firing induced by a depolarizing current step were detected in the two experimental conditions.

In addition, as reported in Table 2, no changes in action potential threshold, amplitude and half width were detected in layer IV spiny neurons from controls and RSL3-treated (5 μM) slices.

**TABLE 2:** Action potential threshold, amplitude and half width measured in layer IV spiny neurons from control and RSL3-treated slices.

	N	Action potential threshold	Action potential amplitude	Action potential half width
Control	12	−50.2 ± 2.2 mV	84.0 ± 4.0 mV	1.2 ± 0.4 ms
RSL3 (5 μM)	12	−50.4 ± 1.8 mV	83.8 ± 4.3 mV	1.2 ± 0.6 ms

The input/output curves obtained in controls and in RSL3-treated slices completely overlapped, All together these results suggest that RSL3 does not affect the passive and active membrane properties of spiny neurons.

**FIGURE 3:**
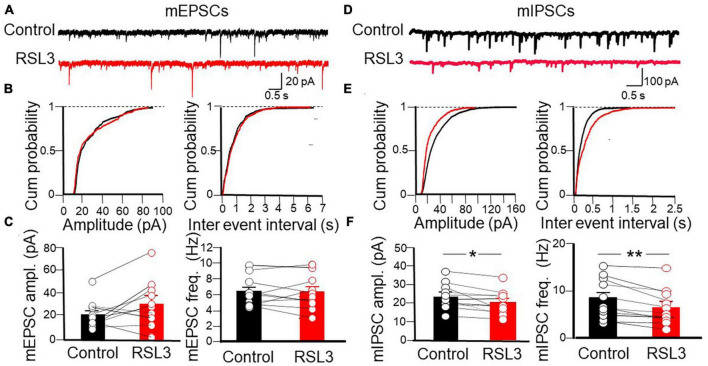
RSL3 induces a significant reduction in frequency and amplitude of mIPSCs but not mEPSCs. **(A)** Sample traces of mEPSCs recorded (in picrotoxin) from layer IV spiny neurons in the absence (black) or in the presence (red) of RSL3 (5 μM). **(B)** Cumulative probability plots of amplitude (left) and inter-event intervals (right) obtained in the absence (black, *n* = 10/5 animals) or in the presence (red, *n* = 10/5 animals) of RSL3. **(C)** Each column represents the mean amplitude (left) and frequency (right) of mEPSCs recorded in the absence (black) or in the presence (red) of RSL3. Bars represent the SEM. Open circles refer to individual values. **(D)** Sample traces of mIPSCs recorded, in the presence of CNQX, from layer IV spiny neurons in the absence (black) or in the presence (red) of RSL3 (5 μM). **(E)** Cumulative probability plots of amplitude (left) and inter-event intervals (right) of mIPSCs obtained in the absence (black, *n* = 11/5 animals) or in the presence (red, *n* = 11/5 animals) of RSL3. Significant reductions in amplitude (*p* < 0.001, K-S test) and frequency (*p* < 0.0001, K-S test) of mIPSCs were found in RSL3-treated slices as compared to controls. **(F)** Each column represents the mean amplitude (left) and frequency (right) of mEPSCs recorded in the absence (black) or in the presence (red) of RSL3. Bars represent the SEM. Open circles refer to individual values. *p < 0.05; **p < 0.005 (paired Student *t*-test).

Next, to assess whether RSL3-induced changes in network excitability involve alterations of excitatory and/or inhibitory synaptic transmission, we recorded spontaneous miniature excitatory and inhibitory postsynaptic currents (mEPSCs and mIPSCs) from layer IV principal cells at −70 mV, in the presence of TTX (1 μM) to block sodium currents and propagated action potentials, picrotoxin (100 μM) or CNQX (10 μM) to block GABA_A_ or AMPA receptors, respectively. Bath application of RSL3 (5 μM) caused a significant reduction in frequency of mIPSCs. This effect was associated with significant changes in mIPSCs amplitude. On average, the frequency of mIPSCs was 8.29 ± 1.34 Hz and 6.31 ± 1.19 Hz before and after application of RSL3, respectively (*p* = 0.002, paired Student-*t*-test) while the amplitude was 23.75 ± 2.17 pA and 20.11 ± 2.02 pA before and after application of RSL3, respectively (*p* = 0.01, paired Student *t*-test). While the reduced frequency of mIPSCs suggests a presynaptic site of action, probably related to a reduced probability of GABA release from GABAergic terminals, the reduced amplitude may depend on both pre and postsynaptic actions, namely, a reduced quantal size or a reduced number of postsynaptic GABA_A_ receptors. No effects on the frequency and amplitude of mEPSCs were detected (Figure 3). On average, the frequency of mEPSCs was 6.22 ± 0.58 Hz and 6.13 ± 0.70 Hz before and after application of RSL3, respectively (*p* = 0.8, paired Student-*t*-test) while the amplitude was 22.3 ± 3.9 pA and 32.7 ± 6.4 pA before and after application of RSL3, respectively (*p* = 0.07, paired Student-*t*-test).

**FIGURE 4:**
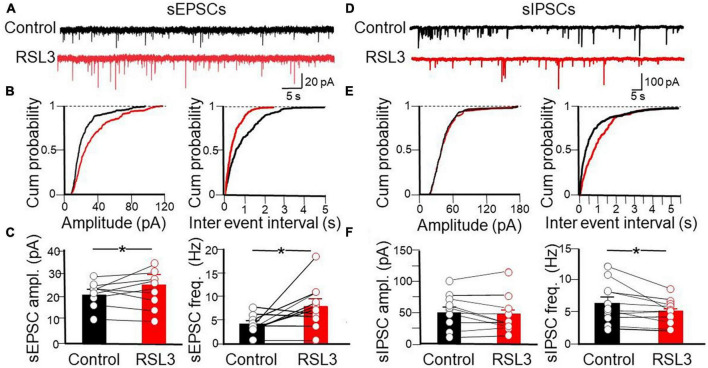
Disinhibitory effect of RSL3 on layer IV spiny neurons. **(A)** Sample traces of sEPSCs recorded (in picrotoxin) from layer IV spiny neurons in the absence (black) or in the presence (red) of RSL3 (5 μM). **(B)** Cumulative probability plots of amplitude (left) and inter-event intervals (right) obtained in the absence (black, *n* = 10/5 animals) or in the presence (red, *n* = 10/5 animals) of RSL3. Significant increases of amplitude (*p* < 0.0001, K-S test) and frequency (*p* < 0.0001, K-S test) of sEPSCs were found in RSL3-treated slices, as compared to controls. **(C)** Each column represents the mean amplitude (left) and frequency (right) of sEPSCs recorded in the absence (black) or in the presence (red) of RSL3. Bars represent the SEM. Open circles refer to individual values. *p < 0.05 (paired Student t-test). **(D)** Sample traces of sIPSCs recorded, in the presence of CNQX, from layer IV spiny neurons in the absence (black) or in the presence (red) of RSL3 (5 μM). **(E)** Cumulative probability plots of amplitude (left) and inter-event intervals (right) of sIPSCs obtained in the absence (black, *n* = 11/4 animals) or in the presence (red, *n* = 11/4 animals) of RSL3. A significant reduction in frequency (*p* < 0.0001, K-S test) but not in amplitude of sIPSCs was found between controls and RSL3-treated slices. **(F)** Each column represents the mean amplitude (left) and frequency (right) of sEPSCs recorded in the absence (black) or in the presence (red) of RSL3. Bars represent the SEM. Open circles refer to individual values. *p < 0.05 (paired Student *t*-test).

To verify whether RSL3, acting mainly on GABAergic interneurons, controls at the network level the excitatory drive to layer IV principal cells, we tested the effect of the drug on spontaneous, action potential-dependent excitatory and inhibitory postsynaptic currents (sEPSCs and sIPSCs), recorded at −70 mV in the presence of picrotoxin or CNQX, respectively (Figure 4). RSL3 (5 μM) induced a significant increase in frequency (from 4.07 ± 0.68 Hz to 8 ± 0.89 Hz, *p* = 0.02, paired Student-*t*-test) and in amplitude (from 21.8 pA ± 1.65 to 25.4 pA ± 1.85, *p* = 0.04, paired Student-*t*-test) of sEPSCs, and a reduction in frequency (from 6.50 ± 0.97 Hz to 5.04 ± 0.58 Hz, *p* = 0.02, paired Student-*t*-test) but not in amplitude of sIPSCs (from 52.59 ± 8.25 pA to 46.59 ± 9.07 pA, *p* = 0.19, paired Student-*t*-test), suggesting that the increase of the excitatory drive to spiny neurons occurred *via* disinhibition.

**FIGURE 5:**
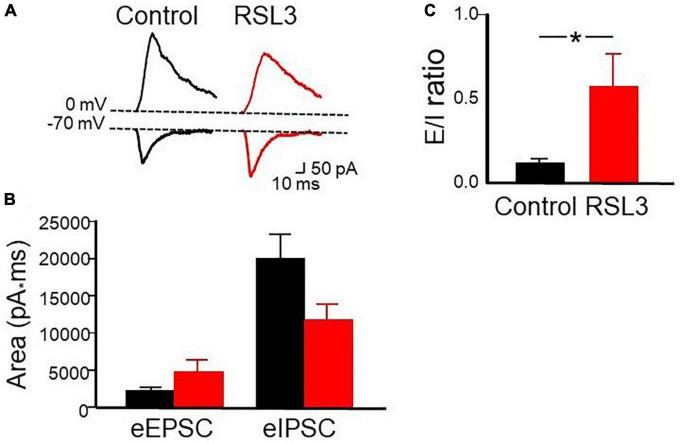
RSL3 affects the excitatory/inhibitory balance. **(A)** Samples of outward (GABAergic) and inward (glutamatergic) synaptic currents evoked in layer IV principal cells by stimulation of afferent fibers at the reversal potential of glutamate (0 mV) and GABA (–70 mV), respectively, in the absence (black) and in the presence (red) of RSL3 (5 μM). Note the decrease in amplitude of the IPSC and the increase in amplitude of the EPSC in RSL3. **(B)** Each column represents the mean area underlying the evoked EPSC or IPSC in the absence (black) or in the presence (red) of RSL3. Bars are the SEM. **(C)** Each column represents the mean E/I ratio (± SEM) observed in control (*n* = 7/3 animals; black) or in RSL3 (*n* = 7/3 animals; red). *p < 0.05 (paired Student’s *t*-test).

In the next series of experiments we monitored the amount of excitation and inhibition received by spiny neurons in response to activation of afferent excitatory and inhibitory fibers through a stimulating electrode localized in layer V. We used a stimulation intensity equal to that necessary to obtain an excitatory postsynaptic current (EPSC) equal to 50% of the maximal. It should be stressed that stimulation of afferent inputs in layer V evokes in spiny neurons, held at their resting membrane potential, an EPSC followed with a short delay by an IPSC. The IPSC is triggered in a feed-forward disynaptic way as demonstrated by its short delay after the EPSC and by its suppression by CNQX (Cellot and Cherubini, 2014). We measured the area underlying the EPSCs and IPSCs recorded in isolation at the reversal potentials of IPSCs (−70 mV) and EPSCs (0 mV), respectively, in control conditions and after treating the slices with RSL3 (5 μM). We calculated the E/I balance by measuring the mean ratio between respective excitatory and inhibitory areas (Figure 5).

As shown in the Figure, respect to control in the presence of RSL3, the Excitatory (E)/inhibitory (I) ratio increased significantly (from 0.119 ± 0.02 to 0.57 ± 0.19; *p* = 0.05, paired Student *t*-test). A proper E/I ratio or E/I balance is thought to be critical for controlling spike rate and information processing. Alterations in the E/I balance within selective brain areas are common to several neurodevelopmental disorders including epilepsy. A reduced inhibition or an excessive excitation may cause an increased signal to noise ratio with consequent neuronal hyper-excitability and seizures (Cherubini et al., 2022).

**FIGURE 6:**
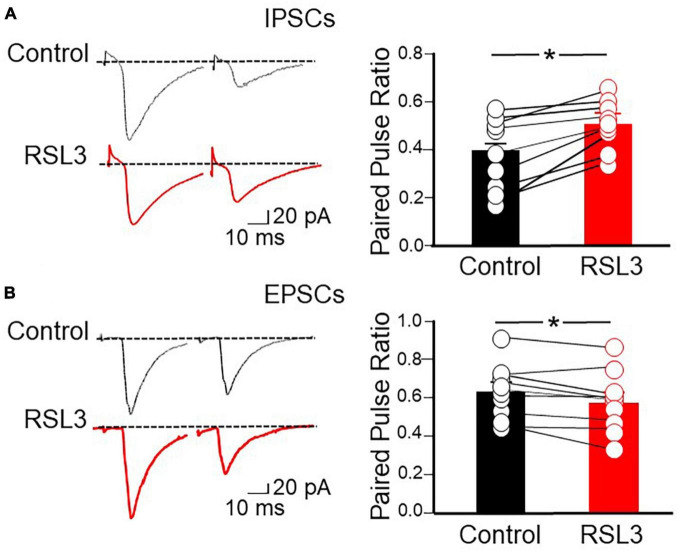
RSL3 reduces the probability of GABA release and enhances that of glutamate release. **(A)** Left: sample traces of IPSCs evoked in layer IV spiny neurons by two stimuli (50 ms apart) delivered to afferent GABAergic inputs (in the presence of CNQX), in the absence (black) or in the presence (red) of RSL3 (5 μM). Right: each column represents the mean ratio between the second and first IPSC in control conditions (black; *n* = 8/3) or in the presence of RSL3 (5 μM, red; *n* = 8/3). Bars are the SEM. Open circles represent individual values. *p < 0.05 (paired Student’s *t*-test). **(B)** Left: sample traces of EPSCs evoked in layer IV spiny neurons by two stimuli (50 ms apart) delivered to afferent glutamatergic inputs (in the presence of picrotoxin), in the absence (black) or in the presence (red) of RSL3 (5 μM). Right: each column represents the mean ratio between the second and first EPSC in control conditions (black; *n* = 9/3) or in the presence of RSL3 (red; *n* = 9/3). Bars are the SEM. Open circles represent individual values; *p < 0.05 (paired Student’s *t*-test).

Furthermore, in additional experiments we determined the probability of GABA and glutamate release measuring the paired pulse ratio (PPR) among two pulses delivered, 50 ms apart, to GABAergic and glutamatergic terminals. We found that RSL3 (5 μM) reduced the probability of GABA release and enhanced that of glutamate release as unveiled by the increase and decrease of PPR, respectively (PPR of IPSCs varied from 0.40 ± 0.05 to 0.51 ± 0.03, *p* = 0.035, paired Student-*t*-test; PPR of EPSCs varied from 0.63 ± 0.04 to 0.58 ± 0.05, *p* = 0.019, paired Student-*t*-test), respectively (Figure 6). These data, in line with those obtained for spontaneous synaptic events in RSL 3-treated slices, suggest that the reduction of GABA release is responsible for the enhancement, *via* disinhibition, of excitatory glutamatergic drive to layer IV spiny neurons.

**FIGURE 7:**
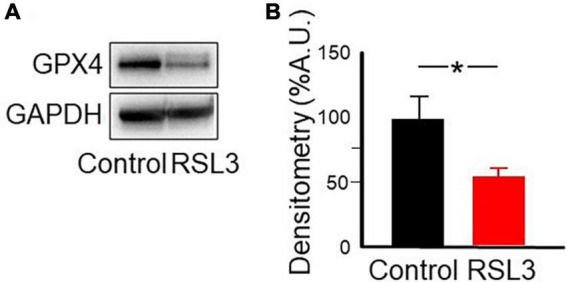
RSL3 acts by reducing GPX4. Representative western blot **(A)** and densitometric analysis **(B)** of GPX4 protein levels in mouse cortical brain slices before and after 2 h treatment with RSL3 (5 μM). Experiments were conducted in triplicates and values expressed as mean ± SD. *p < 0.05 (paired Student *t*-test) as compared to controls.

To evaluate the expression of GPX4 following the RSL3 treatment, cortical slices were analyzed by western blot after 2 h incubation with 5 μM RSL3. As reported in Figure 7, the amount of protein is 45% reduced in RSL3 treated brain slices, respect to untreated ones. RSL3 has been shown to inhibit GPX4 by covalently binding the seleno-cysteine located at the catalytic site of the enzyme (Li et al., 2021). Our data demonstrate that, in brain slices, as a consequence of RSL3 interaction, the inactive GPX4 undergoes degradation.

**FIGURE 8:**
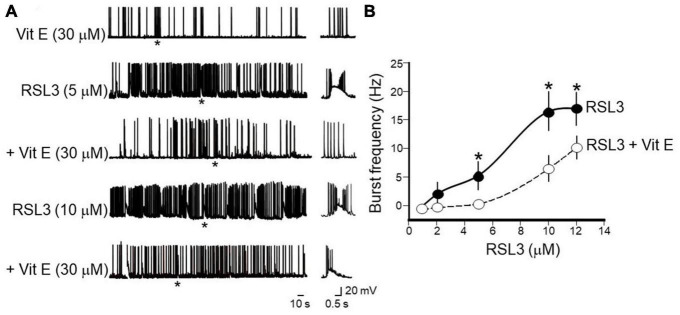
Vitamin E prevents or reduces the frequency of interictal bursts induced by increasing concentrations of RSL3. **(A)** Representative traces showing the beneficial effects of Vitamin E (30 μM) on bursting activity induced at –70 mV in layer IV spiny neurons by RSL3 concentrations of 5 and 10 μM. Note that Vitamin E prevents interictal discharges caused by 5 μM RSL3 (*n* = 6/4 animals) and significantly reduces those produced by 10 μM RSL3 (*n* = 6/4animals). Vitamin E alone (30 μM), did not modify the spontaneous firing of layer IV spiny neurons (see representative trace at the top). Firing or bursting activity marked by an asterisk is shown on the right on an expanded time scale. **(B)** Dose-response curves of burst’s frequency induced by increasing concentrations of RSL3 (continuous line) and by RSL3 plus Vitamin E (30 μM; dashed line). Bars are the SEM. *p < 0.05 (two way ANOVA test).

Vitamin E, known also as tocopherol, is a naturally occurring fat-soluble antioxidant that, by reducing iron accumulation and lipid peroxidation (Conrad et al., 2018) regulates ferroptosis-induced programmed neuronal cell death. Therefore, to see whether inhibiting ferroptosis may provide a novel strategy for preventing or treating epilepsy, in the following experiments, the effects of Vitamin E (30 μM) were tested on interictal bursts induced by increasing concentrations of RSL3 (*n* = 6 slices from 4 animals). To this aim, cortical slices were incubated for 2 h with Vitamin E and then exposed for 15 min to RSL3. Data were compared with those obtained by exposing cortical slices only to RSL3. Pre-treatment with Vitamin E, was able to abolish or reduced the frequency of interictal discharges induced by increasing concentrations of RSL3 (Figure 8). For instance, as shown in the Figure while Vitamin E completely abolished interictal bursts induced by RSL3 (5 μM), it reduced the frequency of those caused by RSL3 (10 μM) from 16.5 ± 4.5 Hz to 9 ± 1.8 Hz. Vitamin E *per se* did not modify spontaneous firing of layer IV spiny neurons.

These data further confirm the crucial role played by ferroptosis in epilepsy and the beneficial effects produced by antioxidant moieties such as Vitamin E, capable of scavenging lipid ROS.

## Discussion

The present study clearly shows that, in mouse cortical slices, the ferroptosis inducer RSL3 produces in layer IV principal cells of the barrel cortex, spontaneous interictal discharges which persist for at least 30 min after wash out of the drug. The RSL3-induced interictal bursts are network-driven events, since they occur simultaneously with field potentials recorded with an extracellular electrode positioned in layer V, close to the apical dendrites of spiny neurons where excitatory inputs, mainly of thalamic origin, make synaptic contacts with layer IV pyramidal cells. Furthermore, in contrast to endogenously generated bursts, RSL3-induced interictal discharges are unaffected by hyperpolarizing the membrane toward more negative values, indicating that they are not dependent on intrinsic membrane properties of the cell. In addition, the observation that RSL3 fails to modify the passive and active membrane properties of principal cells, as demonstrated by similar membrane potential, capacitance and input resistance values as well as spike’s properties, is against their endogenous origin. Furthermore, the similar input/output curves, obtained in control and in RSL3-treated slices, in the presence of synaptic transmission blockers, further support this view.

It is worth mentioning that RSL3-induced bursts are very similar to paroxysmal discharges produced by several convulsants including pentylentetrazol (Bingmann and Speckmann, 1986), kainate (Lévesque and Avoli, 2013), penicillin and bicuculline (Traub and Wong, 1983), high potassium (Rutecki et al., 1985), 4-aminopyridine (Perreault and Avoli, 1991), known to be generated within a polysynaptic circuit. In agreement with most animal models of epilepsy, also in our case, interictal discharges are associated to a significant reduction of GABA_A_-mediated synaptic inhibition. RSL3 acts mainly presynaptically, reducing the probability of GABA release from GABAergic terminals as assessed by the decrease in frequency but not in amplitude of spontaneously occurring inhibitory postsynaptic events. As observed in several animal models of epilepsy (Ribak et al., 1982; Kobayashi and Buckmaster, 2003; Sun et al., 2007), a reduced GABAergic transmission may rely also on a loss of interneurons, known to be more vulnerable than glutamatergic ones (Magloire et al., 2019). Although GABAergic interneurons comprise less than 20% of the entire neuronal population, they play a crucial role in regulating cell excitability in cortical circuits (Pelkey et al., 2017). In our experiments, the reduced GABAergic tone was associated to an increase of action potential-dependent glutamatergic transmission recorded in the absence of tetrodotoxin, suggesting a disinhibitory mechanism. This leads to an excitatory (E)/inhibitory (I) unbalance within a cortical microcircuit. Failure to maintain a proper E/I balance accounts for behavioral deficits observed in several neurological diseases including epilepsy (Cherubini et al., 2022). In particular, the excessive excitatory glutamatergic drive to layer IV pyramidal cells, caused by disinhibition, may lead to an increased signal to noise ratio with consequent enhancement of neuronal excitability. Furthermore, the reduced inhibitory tone to spiny neurons may affect the inhibitory gate that controls sensory information from thalamic afferents and information processing.

The direct interaction of RSL3 with GPX4 leads to a decrease of protein expression, as demonstrated in western blot experiments performed on cortical slices exposed to the drug. In physiological conditions, GPX4 prevents ferroptosis and cell death by neutralizing lipid peroxides and iron overload (Ingold et al., 2017). GPX4 requires as a cofactor glutathione, which is controlled by cysteine, whose levels are regulated by glutamate *via* the cysteine-glutamate antiporter (system Xc). This mediates the uptake of extracellular cysteine in exchange of glutamate (Bridges et al., 2012). Therefore, GPX4 may be involved in maintaining, in physiological conditions, the Excitatory/Inhibitory balance. In the present experiments, the RSL3-induced enhancement of glutamate release may contribute to alter the E/I ratio leading to cell hyper-excitability through inhibition of the cysteine-glutamate antiporter and consequent reduction of glutathione’s production (Dixon et al., 2014).

Glutathione-peroxidase-4 is essential for regulating anti-oxidant and anti-apoptotic activities in pre and early postnatal development. Thus, mutations of the GPX4 gene are responsible for Sedaghatian-type spondylometaphyseal dysplasia (OMIM #250220), a rare pediatric syndrome characterized by severe neurological defects, seizures, and cerebellar hypoplasia (Smith et al., 2014). Furthermore, a partial inactivation of GPX4 activity has been recently unveiled in the blood of children with epilepsy (Petrillo et al., 2021).

It should be stressed that the pre and early postnatal period is characterized by network-driven membrane potential oscillations in the hippocampus and cortex (Ben-Ari et al., 1989; Garaschuk et al., 1998), which are required for the proper wiring of developing circuits (Kasyanov et al., 2004; Mohajerani et al., 2007). This period is also characterized by an excess of free iron and a relative deficiency of powerful scavengers of ROS such as superoxide dismutase and glutathione peroxidase (Buonocore et al., 2001), all conditions that make newborns and particularly preterm infants more susceptible to ferroptosis-dependent forms of epilepsy. Interestingly, seizures refractory to current antiepileptic therapy, constitute the most common manifestation of mitochondrial diseases early in postnatal life, (Kahn-Kirby et al., 2019). As in most mammalian cells, mitochondria are the center of metabolism and an important source of ROS which, as already mentioned, play a key role in the induction of ferroptosis (Gao et al., 2019; Lee et al., 2020).

In the present study the effects of RSL3 on interictal discharges could be reduced or prevented by re-balancing redox homeostasis with Vitamin E, a lipophilic antioxidant able to counteract lipid peroxidation. This vitamin has been shown to exert a neuroprotective action on ferroptosis-dependent pentilenetetrazole-kindled model of epilepsy (Alzoubi et al., 2019; Zhang et al., 2022). Moreover, Vitamin E treatment was reported to attenuate the severity and incidence of seizures and to improve passive avoidance learning and memory in kainate-induced temporal lobe epilepsy in rats (Kiasalari et al., 2016).

It is known that some commonly used antiepileptic drugs may impair the endogenous anti-oxidant ability to prevent oxidative stress (Martinc et al., 2014). Therefore, better understanding the mechanisms by which RSL3 enhances excitability of layer IV cortical neurons is crucial for developing new therapeutic tools to treat ferroptosis-dependent drug-resistant forms of epilepsy.

## Data availability statement

The raw data supporting the conclusions of this article will be made available by the authors, without undue reservation.

## Ethics statement

The animal study was reviewed and approved by European Brain Research Institute.

## Author contributions

MG, FP, SP, FV, NS, and EC designed the experiments. MG performed and analyzed the electrophysiological experiments and prepared most of figures. JD’A, CT, AQ, and SP performed and analyzed the biochemical experiments and revised the literature. FP and EC wrote the manuscript. All authors contributed to the article and approved the submitted version.
